# High Sensing Performance Toward Acetone Vapor Using TiO_2_ Flower-Like Nanomaterials

**DOI:** 10.1186/s11671-022-03721-4

**Published:** 2022-09-02

**Authors:** Weiye Yang, Quanhong Ou, Xueqian Yan, Lei Liu, Shaoyu Liu, Huohuo Chen, Yingkai Liu

**Affiliations:** 1 Yunnan Key Laboratory of Opto-Electronic Information Technology, Yunnan Normal University, Kunming, 650500 China; 2 Institute of Physics and Electronic Information, Yunnan Normal University, Kunming, 650500 China; 3 Key Laboratory of Advanced Technique and Preparation for Renewable Energy Materials, Ministry of Education, Yunnan Normal University, Kunming, 650500 China; 4 Zunyi Medical University, Zunyi, 563000 China

**Keywords:** Flower-like TiO_2_, Hydrothermal method, Acetone, Gas sensing, Medical diagnosis

## Abstract

For real-application gas sensors, high performances (response, selectivity, response/recovery time and stability) are demanded. An effective strategy is applying nanomaterials in gas sensors. In this study, the anatase TiO_2_ flower-like nanomaterials (FLNMs) are prepared through a one-step hydrothermal method which exhibit high-performance toward acetone vapor. TiO_2_ FLNMs sensors property are characterized at optimal working temperature of 330 °C with selectivity (acetone), response (*S* = 33.72 toward 250 ppm acetone), linear dependence (*R*^2^ = 0.9913), response/recovery time (46/24 s toward 250 ppm acetone) and long-term stability (30 days). These demonstrate that TiO_2_ FLNMs get a high performance for acetone sensor. Moreover, the limit of detection of acetone is 0.65 ppm which is lower than that of exhaled air for diabetes (0.8 ppm), indicating that TiO_2_ FLNMs gas sensor gets potential application in medical diagnosis.

## Introduction

Over the last couple of decades, due to tremendous requirements for application in plenty of fields such as industrial security and medical diagnosis environmental protection, gas sensors have attracted enthusiastic interest in academic circle [1, 2]. Acetone is a universally used raw material, which is transparent and colorless volatile organic chemical contained with distinct taste and smell, cleanser and diluent in laboratories and industrial manufacture. It is needed to monitor colorless volatile organic concentrations in the workplace for safety and environment for health owing to their explosive possibility and toxicity [3]. Symptoms such as light-headedness and fatigue may be caused by exposure to a certain concentration of acetone (500–2000 ppm). While the concentrations of acetone arise over 2000 ppm, it can give rise to coma, muscle weakness, nausea and even death [4, 5]. Furthermore, acetone concentration in exhaled air is an important indicator of diabetes [6, 7]. It is reported that the concentration of acetone in exhaled air is higher than 1.8 ppm for diabetic, while it is lower than 0.8 ppm for able-bodied person [8]. Therefore, low concentration detection of acetone is significant for diabetes diagnosis.

Metal oxide semiconductors (MOSs) exhibit outstanding sensing performance due to the plentiful oxygen vacancies, which can be beneficial to gas adsorption. Among the MOSs, titanium dioxide (TiO_2_) is a widely researched n-type MOSs, which has been used in numerous areas such as solar cells [9], electrochemistry [10, 11], photocatalysis [12] and gas sensors [13]. Various kinds of TiO_2_ nanostructures with distinct morphologies and sizes, such as nanowires, nanotubes, nanoparticles and nanobelts have been applied for acetone detecting and displayed high sensing properties [14, 15], whereas several shortcomings like poor stability, low selectivity, low response and long response/recovery times, restricted TiO_2_ nanomaterials in realistic application. To conquer these drawbacks, strategies such as core–shell structures [16], doping [17], functionalize with noble metals [18] and light activation [19] have been demonstrated.

Inspired by these, TiO_2_ flower-like nanomaterials (FLNMs) are successfully prepared through a one-step hydrothermal method, which exhibit high sensing performance for acetone. The TiO_2_ FLNMs’ morphology, crystal structure and elementary composition are analyzed by scanning electron microscope (SEM), transmission electron microscopy (TEM), X-ray diffraction (XRD) and X-ray photoelectron spectroscopy (XPS), respectively. Moreover, the gas properties are well researched and sensing mechanism has also been discussed. These results reveal that TiO_2_ FLNMs display greatly response, stability and response properties to the acetone. TiO_2_ FLNMs shows a high performance in detecting low concentration acetone, which can be used for diabetes diagnosis.

## Experimental Section

### Materials

Titanium films (99.999% purity) are bought from Haiyuan Aluminum Corporation, hydrofluoric acid (HF, 40 wt%) is supplied by Tianjin Chemical Reagent Corporation. Acetone (C_3_H_6_O, 99.5%), ethanol absolute (C_2_H_6_O, 99.7%), benzene (C_6_H_6_, 99.5%), toluene (C_6_H5CH_3_, 99.5%), xylene (C_8_H_10_, 99.0%), methanol (CH_3_OH, 99.5%) and formaldehyde solution (HCHO, 37–40%) are bought from Tianjin Chemical Reagent Corporation. All experimental water is deionized water (18.2 MΩ) in this work. All reagents are purchased without any further purification.

### Sample Preparation

TiO_2_ FLNMs are prepared through a one-step hydrothermal reaction between titanium films and HF, which is mentioned in previous report [20, 21]. Firstly, titanium films (3 cm × 1 cm) are treated by a basic procedure of decontamination. Secondly, titanium films and 10 mmol HF 60 mL are placed to a 100-mL Teflon autoclave, maintaining at the temperature of 110 °C for 6 h. The reaction occurs only on the surface of the titanium film, gray precipitates are scratched off and collected after the autoclave cooling down to room temperature, the final products are washed alternately with absolute ethanol and deionized water for three times. Finally, the specimens are dried at 80 °C and the pure TiO_2_ FLNMs powder is obtained. The mechanism of the formation of titania processes is as follows.

1 $${\text{Ti}} + 6{\text{HF }} \to {\text{H}}_{2} {\text{TiF}}_{6} + 2{\text{H}}_{2} \uparrow$$

2 $${\text{H}}_{2} {\text{TiF}}_{6} + 4{\text{H}}_{2} {\text{O}} \to {\text{Ti}}\left( {{\text{OH}}} \right)_{4} + 6{\text{HF}}$$

3 $${\text{Ti}}\left( {{\text{OH}}} \right)_{4} \to {\text{TiO}}_{2} + 2{\text{H}}_{2} {\text{O}}$$

### Characterizations

The surface morphologies are obtained by scanning electron microscope with an acceleration voltage of 30.0 kV (SEM, Quanta FEG 250, FEI, USA). Transmission electron microscopy (TEM) is observed on a JEM-2100 electron microscope. The crystalline phase of all the specimens is identified by the X-ray diffraction analysis with Cu-Kα1 radiation (*λ* = 1.5405 Å) scanning from 20° to 80° (XRD, DX-1000, Dandong Fangyuan Instrument Co. Ltd., China). X-ray photoelectron spectroscopy (XPS) is measured to reveal chemical valence states and chemical composition, it was performed on an imaging photoelectron spectrometer (Thermo Fisher Scientific, USA) with a monochromatic Al Kα X-ray source. The gas sensing properties are accomplished by CGS-1TP intelligent gas sensing analysis system (Beijing Elite Tech Co., Ltd. China).

### Sensor Preparation

**Fig. 1:**
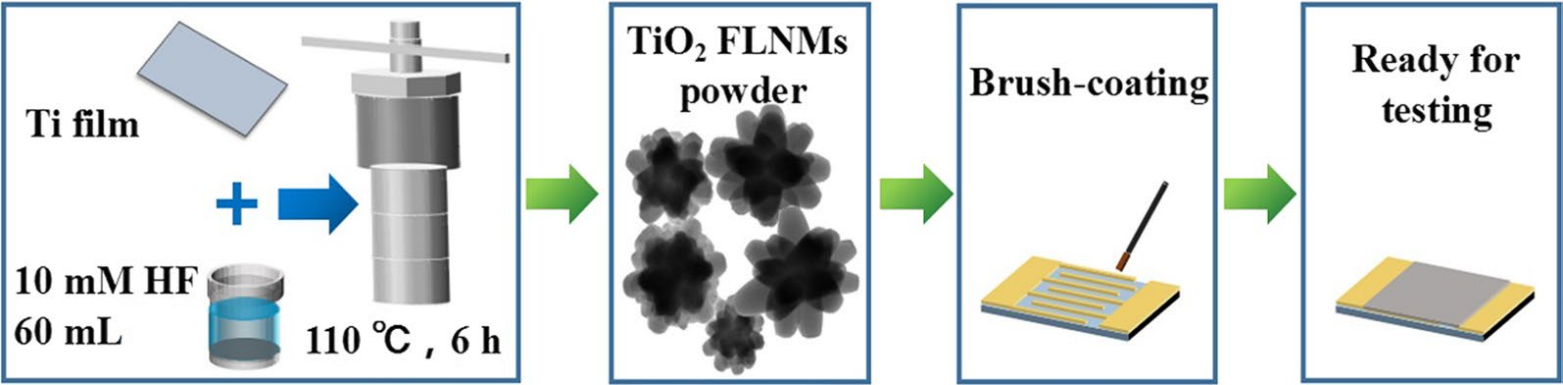
The schematic diagram of TiO_2_ FLNMs sensor preparation and test

The gas sensors are made by a brush-coating technique. Firstly, TiO_2_ FLNMs powder is mixed with deionized water to create a uniform slurry after grinding. Secondly, the paste is coated onto Ag interdigitated electrodes by a paint brush. To increase the stability and repeatability, the prepared sensors are aged at 330 °C for 12 h in ambient air. The related preparation process is displayed in Fig. 1.

The gas sensing measurement are achieved via CGS-1TP intelligent gas sensing analysis system. The microsyringe with the different target liquids is injected into the testing chamber (18 L). Target liquids evaporate into vapor in the chamber. The resistances of TiO_2_ FLNMs sensors change rapidly and reach into a steady value. Then, the air is pumped into testing chamber to restore the sensor resistance to its pretest value. The gas sensor response is defined as *S* = *R*_a_/*R*_g_, which *R*_g_ and *R*_a_ is the resistance under target gas and atmosphere, respectively. The response time/recovery time is defined as the time needed to reach 90% of the saturation S value change upon the target gas or ambient air, respectively.

## Results and Discussion

### Characterization

**Fig. 2:**
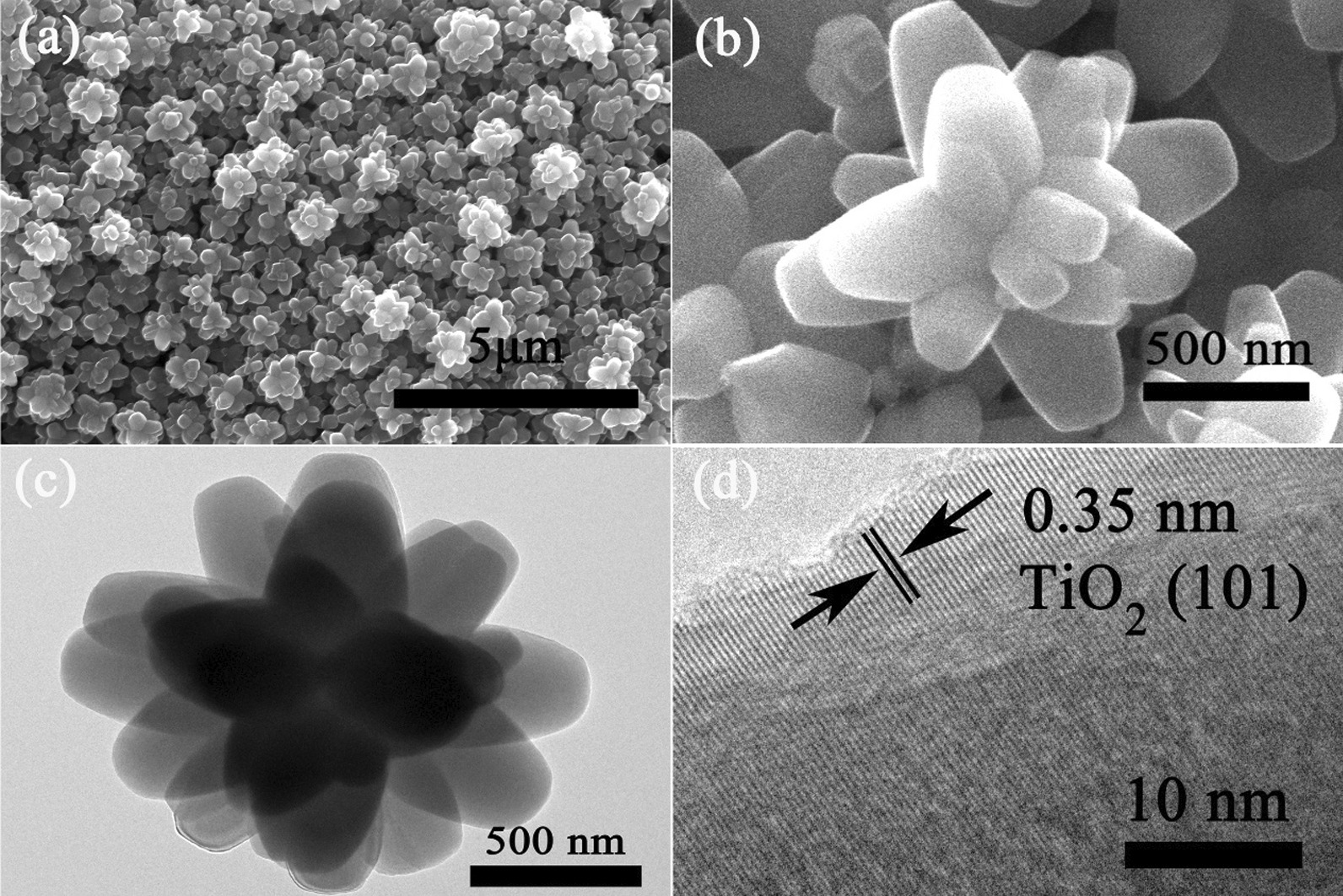
**a**, **b** SEM images of TiO_2_ FLNMs at low and high magnifications, respectively; **c** TEM image of TiO_2_ FLNMs; **d** HRETM image

The surface morphologies and microstructures of TiO_2_ FLNMs are observed by SEM and TEM. SEM images are revealed in Fig. 2a, b. It is evident that TiO_2_ composed of aggregated nanoflowers with size around 0.9–1.7 μm. In order to conform insight of TiO_2_ FLNMs’ microstructures, TEM image is displayed in Fig. 2c. The image exhibits obvious flower-like nanostructure. The crystalline structure of TiO_2_ FLNMs is further demonstrated by high-resolution TEM (HRTEM), as shown in Fig. 2d. The lattice space is 0.35 nm which is well matched with the anatase TiO_2_ (101).

**Fig. 3:**
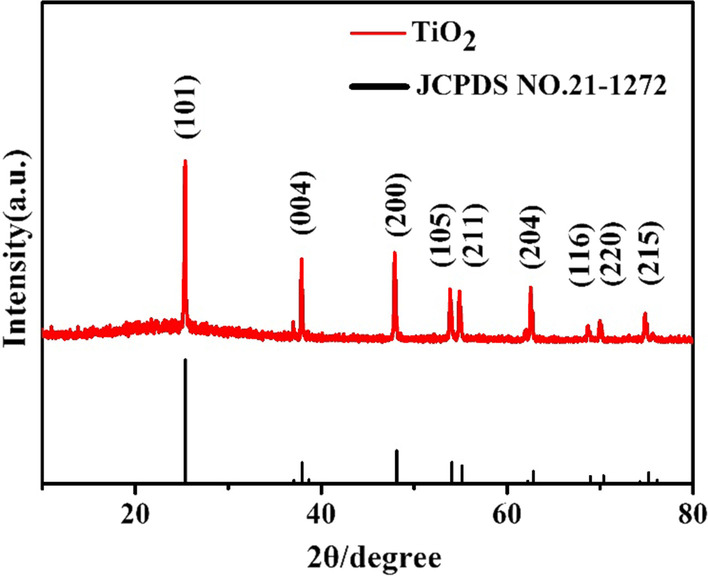
XRD pattern of TiO2 FLNMs

The crystal structure of TiO_2_ FLNMs is also confirmed by XRD, which is portrayed in Fig. 3. It is revealed that the characteristic diffraction peaks is anatase TiO_2_ match with the standard data (JCPDS. 21-1272). The composition phase located at 2*θ* of 25.3°, 37.9°, 48.1°, 54.0°, 55.1°, 62.8°, 68.9°, 70.3° and 75.1° are assigned to (101), (004), (200), (105), (211), (204), (116), (220) and (215) planes of anatase phase.

**Fig. 4:**
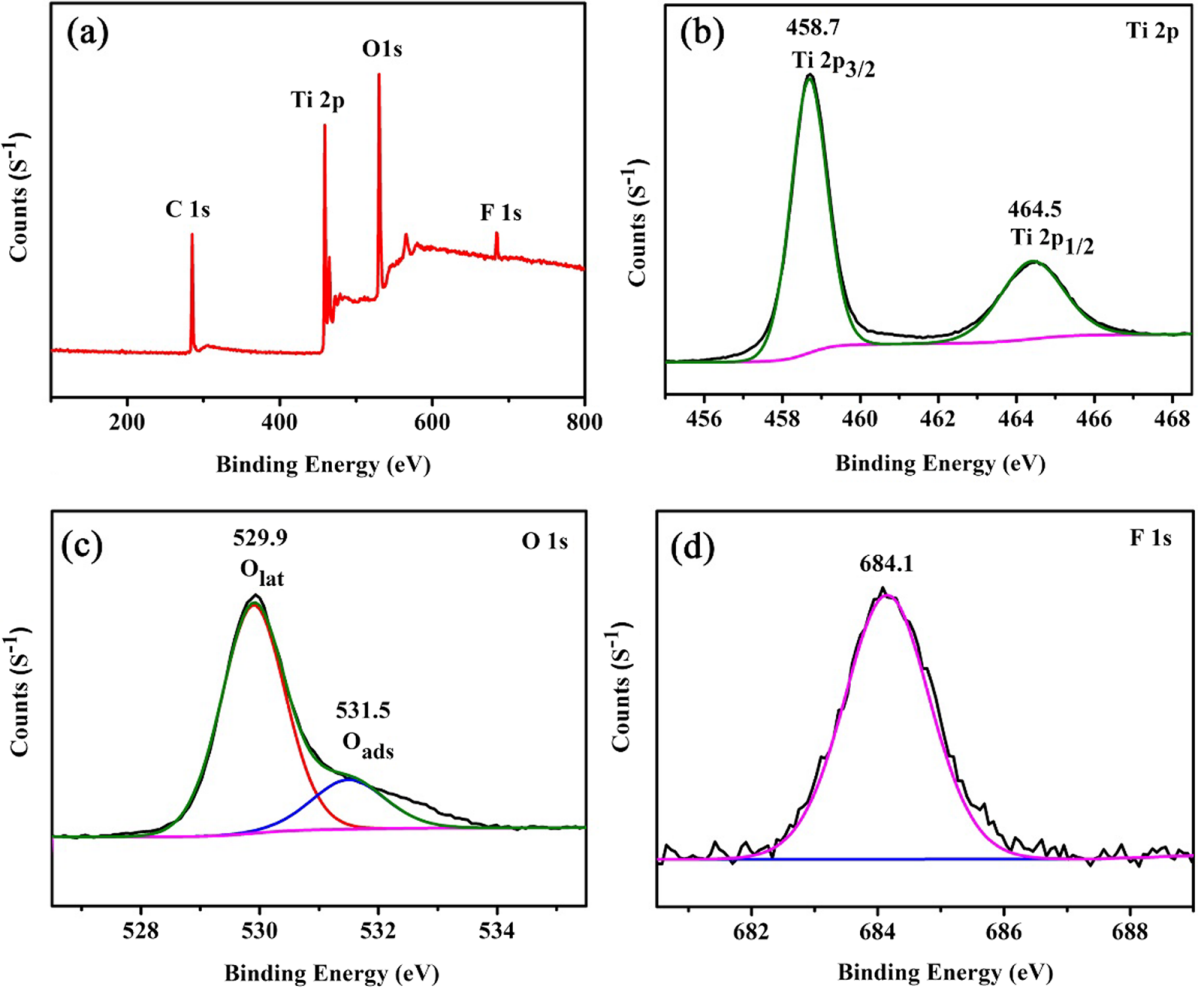
XPS analysis of TiO2 FLNMs: **a** the full XPS spectrum, **b** Ti 2*p* spectrum, **c** O1*s* spectrum, **d** F 1*s* spectrum

XPS analysis is applied to confirm the surface elemental chemical states and elementary compositions. Figure 4a shows the full-scale XPS survey scan spectra. It reveals that TiO_2_ FLNMs compose of Ti, O and F elements, indicating that there are no other impurities, F element is the residual of HF originating from synthetic process. C 1*s* at 284.8 eV is used to the calibration peak. The peaks centered at 458.7 eV and 464.5 eV are corresponding to the spin–orbit split lines of Ti^4+^ 2*p*_3/2_ and Ti^4+^ 2*p*_1/2_, respectively. (Fig. 4b) [22, 23] Fig. 4c displays peaks around at 529.9 eV and 531.5 eV can be assigned to crystal lattice oxygen ions (*O*_lat_) and surface adsorbed oxygen ions (*O*_ads_). The adsorbed oxygen ions play a significant role in gas sensing property [24–26]. Figure 4d reveals a peak at 684.1 eV which is derived from the adsorbed F on the TiO_2_ surface [27].

**Fig. 5:**
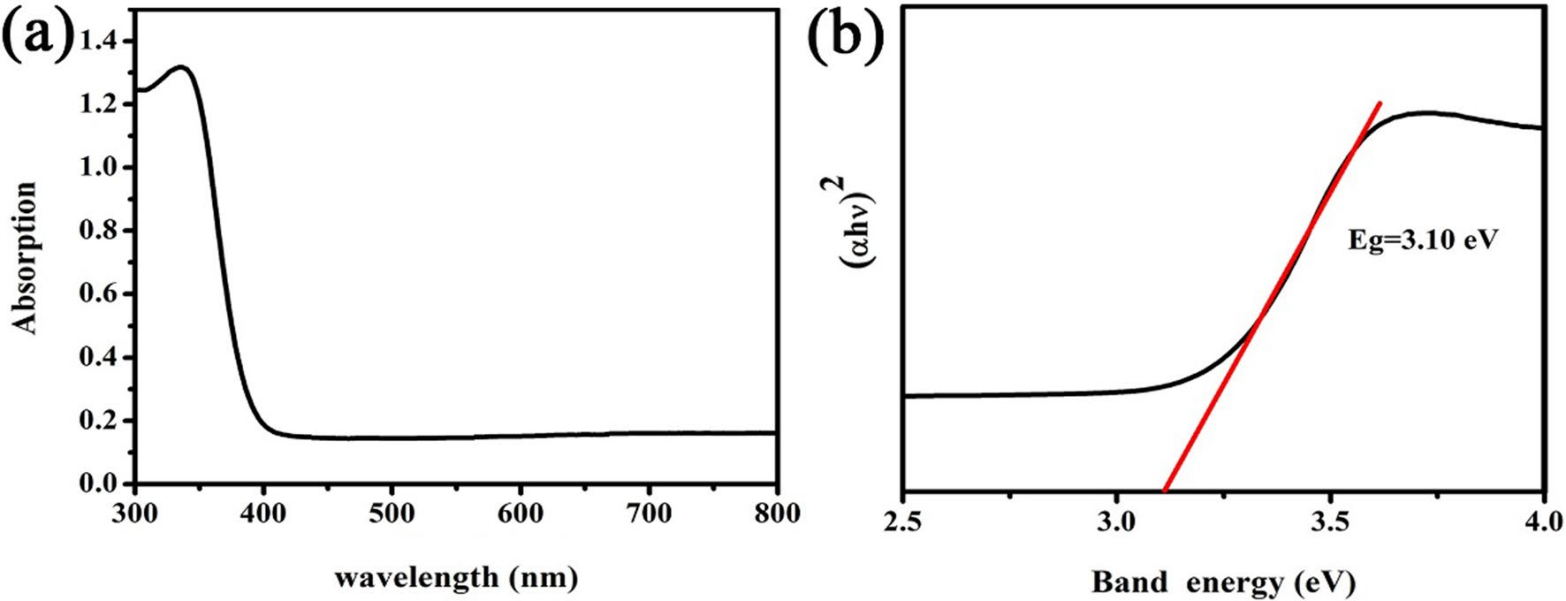
**a** UV–vis adsorption spectrum of TiO_2_ FLNMs; **b** relationship between $\left( {\alpha h\nu } \right)^{2}$ ~ $h\nu$

It is seen from Fig. 5a that the TiO_2_ FLNMs has a strong absorption in the range of 300–400 nm with absorption edge of 400 nm. Its band gap is calculated by the following equation.

4 $$\alpha h\nu = A\left( {h\nu - E_{{\text{g}}} } \right)^{2}$$

The band gap (*E*g) of the TiO_2_ FLNMs is 3.10 eV, being smaller than that of anatase (3.20 eV), as shown in Fig. 5b. It is reported that F residual have an effect on band gap. It is beneficial to reduce the band gap, facilitate electron transition and improve material properties [28, 29].

### Acetone Sensing Properties

**Fig. 6:**
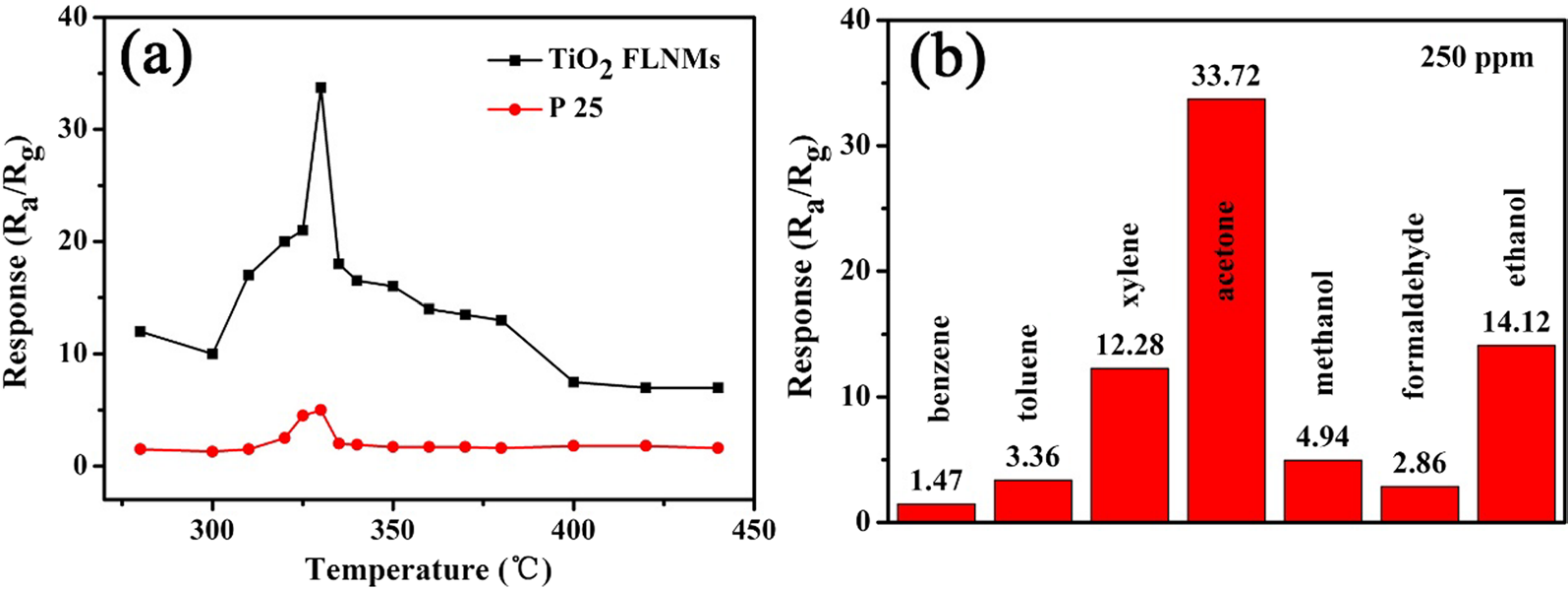
**a** Response of TiO_2_ FLNMs an Degussa P25 powder toward 250 ppm acetone at different operating temperature; **b** selectivity test of TiO_2_ FLNMs gas sensor (250 ppm)

To assess applicability of TiO_2_ FLNMs sensor for acetone, some fundamental parameters are studied. As we all know, the working temperature is a significant parameter for gas sensors owning to its vigorously affect surface reaction and chemisorption between oxygen and target gas molecules. The most excellent response can be reached at an optimum working temperature when the absorption/desorption achieve equilibrium. To find the optimal working temperature, TiO_2_ FLNMs gas sensors are tested under temperatures range from 280 to 440 °C with a concentration of 250 ppm acetone ambient, while the gas sensor prepared with Degussa P25 is also tested at the same condition, as shown in Fig. 6a. It is clearly that the optimal working temperature of TiO2 FLNMs and Degussa P25 both are 330 °C. Thus, all the subsequent experiments are carried out at the optimal working temperature of 330 °C. Furthermore, response of Degussa P25 gas sensors is apparently lower-ranking to TiO_2_ FLNMs gas sensor, whereas its response only can achieve 5.0 for 250 ppm acetone and the response of TiO_2_ FLNMs is 33.72 in the same circumstance. It indicates that TiO_2_ FLNMs show better gas sensing property than commercial Degussa P25. Selectivity represents anti-interference capability to special gases. Thus, the selectivity of TiO_2_ FLNMs is analyzed through exposing to 250 ppm benzene, toluene, xylene, acetone, methanol, formaldehyde and ethanol under optimum working temperature, respectively, as revealed in Fig. 6b. It is clearly displayed that the response toward benzene, toluene, xylene, methanol, formaldehyde and ethanol (1.47, 3.36, 12.28, 4.94, 2.86 and 14.12) is much lower than that of acetone (33.72). It indicates that TiO_2_ FLNMs sensor shows comparatively selectivity for acetone.

**Fig. 7:**
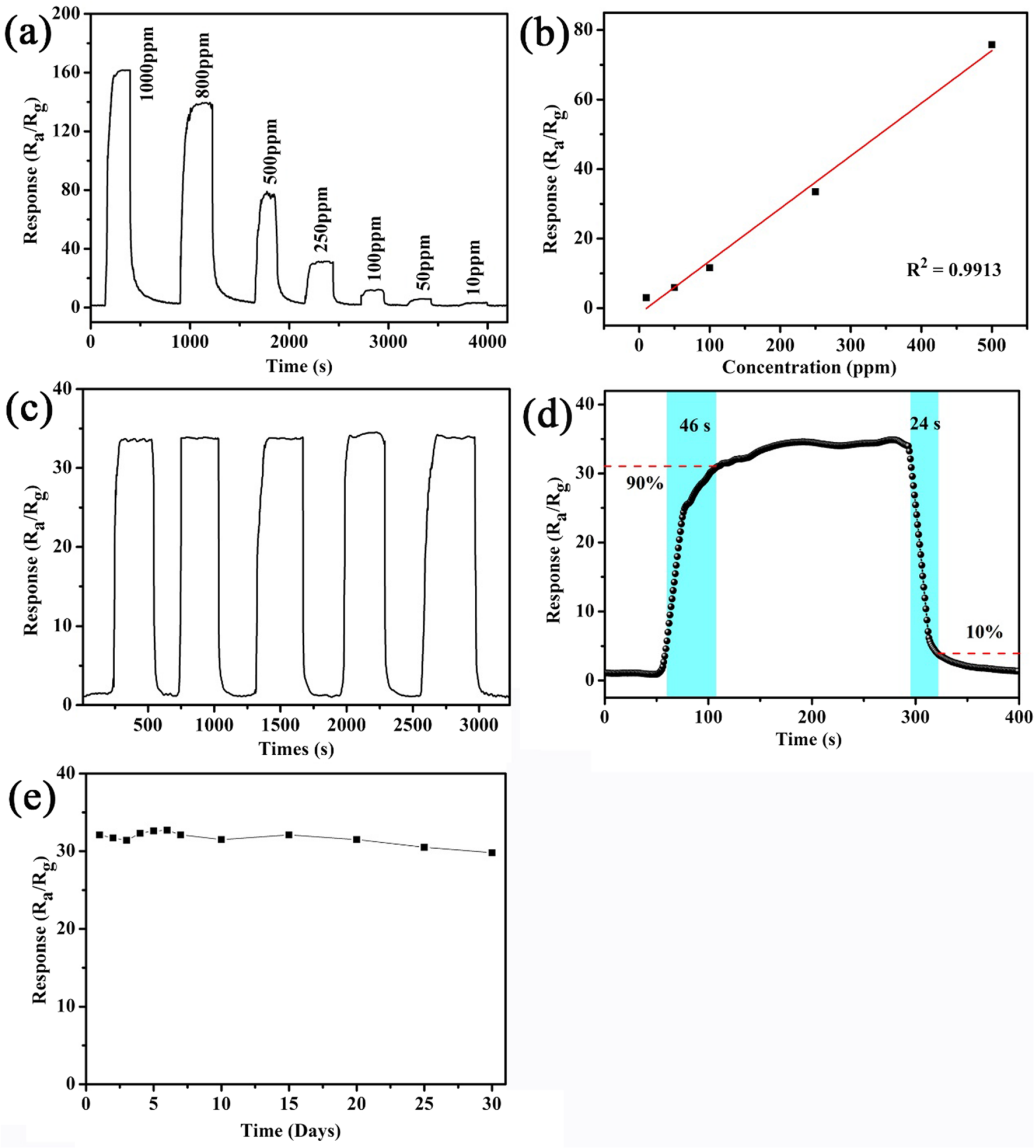
**a** TiO_2_ FLNMs sensing curves versus different concentration (10–1000 ppm) of acetone at 330 °C; **b** the linear response of acetone concentration (10–500 ppm); **c** the reproducibility testing with five cycles; **d** the response/recovery behaviors of TiO_2_ FLNMs; **e** the long-term stability test

The dynamic response–recover curves of TiO_2_ FLNMs sensors versus concentration from 10 to 1000 ppm of acetone and ambient air at optimal working temperature are displayed in Fig. 7a. It is revealed that response is linear relationship in assessing scope. The response of TiO_2_ FLNMs linearly increases with the increment of acetone concentration. The linear response dependence on acetone concentration is also studied in Fig. 7b. It shows that the fitting curves versus concentration of acetone toward 10–500 ppm, the correlated coefficients (*R*^2^) is 0.9913 indicating an outstanding linear-dependent relationship between the response and concentration. Another important parameters limit of detection (LOD) are also calculated [30–32]. The LOD of acetone is 0.65 ppm which is lower than the detection limit for diabetes in exhaled air. Therefore, TiO_2_ FLNMs gas sensor gets potential application in medical diagnosis.

Repeatability is another significant factor to assess the reliability of the pre-prepared sensor. To evaluate the repeatability of TiO_2_ FLNMs sensor, the dynamic sensing toward 250 ppm acetone is measured for 5 cycles (seen in Fig. 7c). It reveals that its response to 250 ppm acetone has no distinct fluctuations, indicating excellent performance of repeatability. Such a high repeatability can be ascribed to the induction of ultra-stable TiO_2_ to enhance its sensing stability. Figure 7d displays the response/recovery time of TiO_2_ FLNMs. It reveals that the response/recovery time are 46 and 23 s toward 250 ppm acetone, respectively. It indicates that TiO_2_ FLNMs sensor exhibits excellent adsorption and desorption property in acetone. This probable reason is that the target molecules are faster and easier dissociated onto the TiO_2_ FLNMs’ surface, leading to a fast decline in the O^2−^ ions concentration on the surface of TiO2 FLNMs and a rapid increase in the electron concentration, which displays an quick response.

In realistic applications, the long-term stability should be examined. TiO_2_ FLNMs gas sensors’ response over 30 days is assessed at the optimal working temperature (seen in Fig. 7e). It illustrates that the responses only have a minor change, which is less than 7.17% of its initial rate. It indicates excellent stability of TiO_2_ FLNMs gas sensor. To conform the brilliant sensing performance of TiO_2_ FLNMs sensor, a comparison of previous reports and this work on acetone is depicted in Table 1, it further demonstrates that TiO_2_ FLNMs represent high acetone sensing performance in this work.

**Table 1:** Comparison of various TiO_2_ nanostructures toward acetone gas sensing performances

Sensing materials	Working temp**e**rature (°C)	Concentration (ppm)	Response (*R*_a_/*R*_g_)	Response/recover time (s)	Reference
Nanoporous TiO_2_	370	500	25.97	13/8	[25]
TiO_2_ nanoparticles	400	200	7.5	240/180	[33]
Nano nanotube	150	1000	2.08	21/38	[34]
TiO_2_ nanorods	500	300	13	12/6	[35]
TiO_2_ nanoflowers	280	200	~ 7	< 50/< 100	[36]
Ag-TiO_2_ nanobelts	260	500	28.25	6/8	[24]
Ag-TiO_2_ nanospheres	350	500	29.1	1/47	[14]
TiO_2_ nanoparticles	270	500	9.19	10/9	[37]
TiO_2_ microsphere	320	100	6.9	–	[38]
Brookite TiO_2_	320	100	2.3	3/183	[39]
TiO_2_ FLNMs	330	250	33.72	46/24	This work

### Acetone Sensing Mechanism

**Fig. 8:**
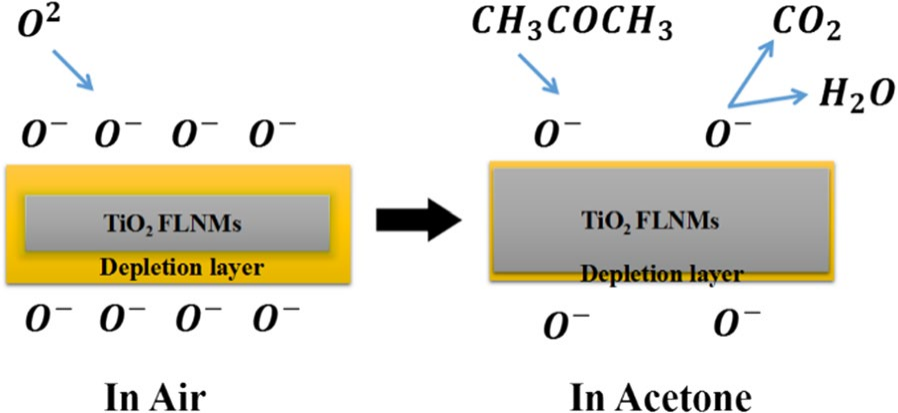
Sensing mechanism of TiO_2_ FLNMs toward ambient air and acetone gas

TiO_2_ is an n-type characteristic semiconductor, while TiO_2_ FLNMs sensors are exposed to the acetone vapor, the resistances declined quickly, implying of n-type semiconductor properties. Gas sensing properties rely on the change of surface occupation. As reported by Wolkenstein’s model for semiconductors [40], we suggest a correspondent model for TiO_2_ FLNMs, as schematically depicted in Fig. 8. In ambient air, oxygen adsorbs onto the surface of TiO_2_ FLNMs, and electron transfers from conduction band to the oxygen molecules to create a variety of oxygen ions with distinct valence states (O_2ads_^−^, O_ads_^−^, O^2−^_ads_), which gives rise to a thick depletion layer’s formation and resulting in a high resistance of the sensor [41, 42]. When TiO_2_ FLNMs sensors are exposed to target gas, the reductive gas responds with the oxygen adsorbed on TiO_2_ FLNMs surface. The electrons are then freed back to the conduction band of semiconductor, resulting in a lower potential barrier and a thinner depletion layer [43]. This procedure leads to a reduction of resistance and can be expressed by the following equations [35]:

5 $${\text{O}}_{2} \left( {{\text{gas}}} \right) \leftrightarrow {\text{O}}_{2} \left( {{\text{ads}}} \right)$$

6 $${\text{O}}_{2} \left( {{\text{ads}}} \right) + {\text{e}}^{ - } \leftrightarrow {\text{O}}_{2}^{ - } \left( {{\text{ads}}} \right)$$

7 $${\text{O}}_{2}^{ - } \left( {{\text{ads}}} \right) + {\text{e}}^{ - } \leftrightarrow 2{\text{O}}^{ - } \left( {{\text{ads}}} \right)$$

8 $${\text{O}}^{ - } \left( {{\text{ads}}} \right) + {\text{e}}^{ - } \leftrightarrow {\text{O}}^{2 - } \left( {{\text{ads}}} \right)$$

9 $${\text{CH}}_{3} {\text{COCH}}_{3} \left( {{\text{gas}}} \right) \leftrightarrow {\text{CH}}_{3} {\text{COCH}}_{3} \left( {{\text{ads}}} \right)$$

10 $${\text{CH}}_{3} {\text{COCH}}_{3} \left( {{\text{gas}}} \right) + 8{\text{O}}^{ - } \leftrightarrow 3{\text{CO}}_{2} \left( {{\text{gas}}} \right) + 3{\text{H}}_{2} {\text{O}}\left( {{\text{gas}}} \right) + 8{\text{e}}^{ - }$$

11 $${\text{CH}}_{3} {\text{COCH}}_{3} \left( {{\text{ads}}} \right) + 4{\text{O}}_{2}^{ - } \left( {{\text{ads}}} \right) \leftrightarrow 3{\text{CO}}_{2} \left( {{\text{gas}}} \right) + 3{\text{H}}_{2} {\text{O}}\left( {{\text{gas}}} \right) + 4{\text{e}}^{ - }$$

## Conclusion

In this work, the newly anatase TiO_2_ FLNMs are successfully prepared though a one-step hydrothermal method, which are composed of uniformly flower like nanostructure with size around 0.9–1.7 μm. It is found that this nanomaterials enable a high-performance toward acetone. The results show high selectivity and response, great linear dependence, excellent repeatability, good response/recovery time and long-term stability at an optimum temperature of 330 °C. Interestingly, the LOD of acetone is 0.65 ppm which is lower than that of in exhaled air for diabetes. These results demonstrate a great potential for medical diagnosis. This study paves a new way of using TiO_2_ FLNMs for acetone sensor.

## Data Availability

All data generated or analyzed during this study are included in this published article.
